# Emerging roles of extracellular vesicles in normal and malignant hematopoiesis

**DOI:** 10.1172/JCI160840

**Published:** 2022-09-15

**Authors:** Guohuan Sun, Quan Gu, Junke Zheng, Hui Cheng, Tao Cheng

**Affiliations:** 1 State Key Laboratory of Experimental Hematology, Haihe Laboratory of Cell Ecosystem, National Clinical Research Center for Blood Diseases, Institute of Hematology and Blood Diseases Hospital, Chinese Academy of Medical Sciences and Peking Union Medical College, Tianjin, China.; 2 Hongqiao International Institute of Medicine, Shanghai Tongren Hospital, Key Laboratory of Cell Differentiation and Apoptosis of Chinese Ministry of Education, Shanghai Jiao Tong University School of Medicine, Shanghai, China.; 3 Center for Stem Cell Medicine, Chinese Academy of Medical Sciences, Tianjin, China.; 4 Department of Stem Cell and Regenerative Medicine, Peking Union Medical College, Tianjin, China.

## Abstract

Hematopoietic stem cells, regulated by their microenvironment (or “niche”), sustain the production of mature blood and immune cells. Leukemia cells remodel the microenvironment to enhance their survival, which is accompanied by the loss of support for normal hematopoiesis in hematologic malignancies. Extracellular vesicles (EVs) mediate intercellular communication in physiological and pathological conditions, and deciphering their functions in cell-cell interactions in the ecosystem can highlight potential therapeutic targets. In this Review, we illustrate the utility of EVs derived from various cell types, focusing on the biological molecules they contain and the behavioral alterations they can induce in recipient cells. We also discuss the potential for clinical application in hematologic malignancies, including EV-based therapeutic regimens, drug delivery via EVs, and the use of EVs (or their cargoes) as biomarkers.

Hematopoiesis occurs in a complex ecosystem where both cellular and noncellular components interact with each other to produce all blood and immune cells in hematopoietic organs under homeostasis or stress conditions. Hematopoietic stem cells (HSCs), with self-renewal and multilineage differentiation capacity, can differentiate into hematopoietic progenitor cells, and further produce mature blood cells to construct the hematopoietic hierarchy; therefore, the normal behavior of HSCs is the foundation of hematopoietic homeostasis. HSCs are protected and supported by a specific microenvironment, termed the hematopoietic niche. Malignant cells can remodel niche cells to create a self-sustaining disease niche that favors malignant cell growth, while suppressing normal hematopoiesis (1, 2). Our understanding of niche components and their roles in normal hematopoiesis and hematopoietic disorders has improved dramatically in recent decades (3, 4). It is now clear that active intercellular communication between HSCs and niche cells underpins HSC function. Despite differences in the immunophenotypical surface markers of human and murine HSCs, these cells possess similar functions and are tightly controlled by their microenvironment (5, 6).

Extracellular vesicles (EVs) play roles in intercellular communication. After their release from parental cells, EVs carry biological cargoes into body fluids. Their subsequent recognition and uptake result in alterations to recipient cell behavior. EV biogenesis, heterogeneity, and regulation are continuous and strictly organized processes that have been reviewed recently (7). Technical limitations have hindered identification of the precise subcellular origin of EVs, leading to overuse of the term “exosome” (8). Hence, we refer to vesicles as EVs throughout this Review. We discuss the biological roles of EVs in normal hematopoiesis and hematopoietic disorders, focusing on the functions of EVs derived from different cell components, to provide a comprehensive overview of this bidirectional intercellular communication system.

## Functions of EVs in normal hematopoiesis

### EVs promote ex vivo expansion of hematopoietic stem/progenitor cells

Hematopoietic stem cell transplantation (HSCT) has been applied successfully to treat hematopoietic diseases and immune disorders for several decades (9). Expanding hematopoietic stem/progenitor cells (HSPCs) ex vivo without compromising their self-renewal capacity would greatly improve the efficacy of HSCT in clinical practice (10), and various studies have demonstrated the value of EVs in achieving this aim. Murine embryonic stem cell–derived (ESC-derived) EVs were used to efficiently expand murine Lineage^–^cKit^+^Sca1^+^ HSPCs via a mechanism that involved upregulation of the expression of stemness-related genes (*Scl*, *HoxB4*, and *Gata2*) (11). Furthermore, mesenchymal stromal cell–derived (MSC-derived) EVs promoted the expansion of mouse myeloid-biased multipotent progenitors via a TLR-engaged mechanism (12). Similarly, microRNA-29a–containing (miR-29a–containing) osteoblast-derived EVs increased human cord blood (CB) CD34^+^ HSPC expansion in vitro and differentiation capacity in vivo by controlling the expression of proliferation-related genes (13). A recent study demonstrated that EVs from fetal calvaria osteoblasts provide better support for normal CB HSPCs than those from fetal limb–derived osteoblasts or human adult bone marrow–derived (BM-derived) MSCs (14). In general, these observations highlight the potential utility of EVs for the expansion of HSPCs ex vivo. For example, EVs isolated from fetal liver, which is a known site of HSC expansion, may effectively expand adult HSCs. Importantly, understanding how EVs contribute to the regulation of HSPC function is necessary to achieve effective and efficient expansion of HSPCs in vitro.

### Roles of EVs in adult HSPC behavior

**Figure 1:**
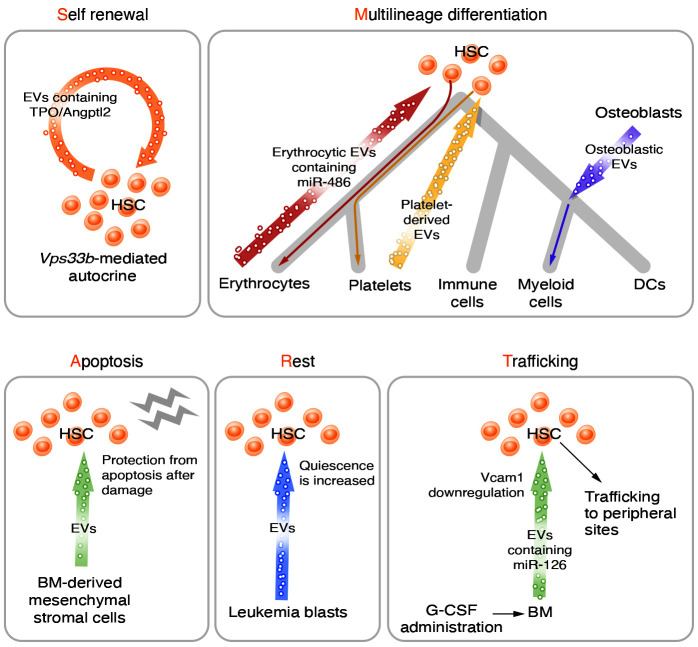
EVs in normal hematopoiesis. EVs derived from various cell types regulate the “SMART” physiological properties of HSCs, spanning self-renewal, multilineage differentiation, apoptosis, rest, and trafficking.

The “SMART” physiological properties of HSCs, spanning self-renewal, maturation, apoptosis, resting, and trafficking, are strictly regulated to ensure homeostasis under steady-state and stress conditions (15, 16). Studies have confirmed the involvement of EVs derived from several cell types in the regulation of HSCs or HSPCs (Figure 1 and Table 1).

#### Self-renewal.

The HSC pool is maintained as a result of their self-renewal capacity (3), and EVs appear to be involved in this process. Blocking EV secretion from HSCs themselves in *Vps33b*-knockout mice dramatically attenuated their self-renewal and repopulating activity. EVs contained stemness-related proteins, such as ANGPTL2, ANGPTL3, and TPO, which contributed to the maintenance of EV-mediated stemness (17). Niche components have also been reported to participate in regulating HSC self-renewal via EVs. Hypoxia-preconditioned MSC-EVs increased the self-renewal capacity of human CB CD133^+^ HSPCs (18). Aged murine MSCs exhibited activated AKT signaling, and their EVs had decreased levels of autophagy-related mRNAs. Furthermore, inhibition of AKT in aged MSCs increased the levels of autophagy-related mRNAs carried by EVs. Incubation with these “rescued” EVs facilitated murine HSC reconstitution in transplantation, indicating the enhancement of HSC self-renewal (19). These findings have offered new options for enhancing in vivo engraftment of HSCs, which has been a long-standing bottleneck in this field.

#### Multilineage differentiation.

EVs derived from hematopoietic and non-hematopoietic cells have been reported to be involved in HSPC differentiation. Coculturing with megakaryocyte-EVs (MK-EVs) promoted the differentiation of human HSPCs into functional MKs (20, 21). During mouse acute liver injury, platelet-derived EVs were found to drive HSPCs toward a megakaryocytic fate (22). Moreover, TLR2-induced MK-EVs promoted MK maturation of a human megakaryocytic cell line (Dami) by increasing cytokine production (23). A similar phenomenon was observed in erythroid differentiation, with exposure of a human erythroleukemia cell line (TF-1) to hypoxia leading to increased EV secretion. These miR-486–containing EVs stimulated proliferation and erythroid differentiation of human CD34^+^ HSPCs, potentially by targeting *Sirt1* (24). A recent study revealed that osteoblastic EVs loaded with transfer RNA–derived stress-induced RNAs (tiRNAs) were preferentially transferred to murine BM granulocyte-macrophage progenitors, resulting in increased protein translation, cell proliferation, and myeloid differentiation in vivo. Stress-modulated transfer of tiRNA-loaded EVs improved hematopoietic recovery from genotoxic injury and prolonged host survival (25). This study offered solid evidence of in vivo EV transfer between BM stroma cells and hematopoietic cells. However, as the indicators of the EV transfer were GFP proteins driven by specific promoters (Ocn-GFP, labeling osteoblasts), the evidence was indirect. Therefore, the use of more definitive EV-labeling reporter tools is required to obtain more direct and quantifiable evidence of EV transfer. For example, by crossing of CD63-GFP^fl/fl^ mice with specific Cre mice, the EVs from the Cre^+^ cells can be labeled as GFP^+^ (26).

#### Apoptosis.

Several studies have provided evidence that MSC-EVs protect HSPCs from apoptosis under various stress conditions. Dental pulp stem cell–EV treatment mitigated apoptosis of a murine myeloid progenitor cell line (FDC-P1) in vitro (27). Injection of MSC-EVs significantly restored murine HSC engraftment capacity after irradiation, potentially by reversing the growth inhibition, DNA damage, and apoptosis induced by irradiation (28). Another report confirmed that MSC-EVs could support human HSC recovery in vitro and the long-term survival of recipients in vivo (29). Incorporating human BM MSC-EVs into CD34^+^ cells induced downregulation of proapoptotic genes and a significant decrease in apoptosis (30). Similarly, EVs released by AFT024, a murine fetal liver–derived stromal cell line, modulated the gene expression pattern of HSPCs and protected them from apoptosis (31). These findings indicate the potential of stromal cell–derived EVs as an antiapoptotic treatment.

#### Resting (quiescence).

Abdelhamed and colleagues described the ability of leukemia-blast–derived EVs to enter murine HSCs (Lineage^–^cKit^+^Sca1^+^CD150^+^CD48^–^), resulting in suppressed protein synthesis and increased cell quiescence, thereby demonstrating the reversibility of murine HSPC dysfunction under leukemia stress (32). However, whether niche cell–derived EVs contribute to the maintenance of HSC quiescence in vivo under physiological conditions remains to be clarified.

#### Trafficking.

Administration of granulocyte colony-stimulating factor (G-CSF) promoted the accumulation of EVs containing miR-126 in the BM extracellular compartment, resulting in reduced VCAM1 expression by murine HSPCs (Lineage^–^cKit^+^Sca1^+^). This observation implicated miR-126–containing EVs in the regulation of HSPC trafficking between the BM and peripheral sites (33). Later research demonstrated that MSC-EV administration downregulated the expression of *Cxcl12*, *Scf*, and *Vcam1* while enhancing G-CSF–induced HSPC mobilization to a similar degree to that promoted by MSCs and G-CSF, thus indicating that the ability of MSCs to affect HSC trafficking is mediated by EVs (34).

Research on EVs has broadened our understanding of their ability to support and regulate hematopoietic processes. Given the gaps in our knowledge, it is unclear whether murine hematopoiesis universally reflects the human process. The mechanisms underlying this process and strategies to improve the efficacy of EV treatments require further investigation. Employing genetic mouse models could offer direct evidence of the function of EVs derived from a certain cell type. To date, the majority of EV-related research has been based on human CD34^+^ or murine Lineage^–^cKit^+^Sca1^+^ cell populations (HSPCs). Since HSPC populations are heterogeneous (35), EV treatment combined with well-defined immunophenotype analysis as well as single-cell multi-omics studies will reveal unrecognized mechanisms. Moreover, the functions of EVs under pathological conditions should be emphasized, as they may regulate disease processes and serve as potential therapeutic targets.

## Functions of EVs in hematologic malignancies

Hematologic malignancies (HMs) represent a heterogeneous group of hematopoietic neoplasms commonly characterized by the abnormal production of blood cells. It is possible that the shelter provided to malignant cells by the cancer-modulated niche contributes to refractory cases and relapse (36). Exploring the exact alterations of the “hijacked” niche and how these promote disease progression could shed light on potential new cancer therapies. Indeed, dissecting the intercellular communication networks among malignant cells, normal cells, and their surrounding microenvironment could provide valuable information on the optimal way to target tumor-permissive niches. Here, we discuss the roles of EVs in HMs, focusing on the cargoes transferred, the genes that are regulated, and how the biological behaviors of recipient cells are altered, as well as the mechanisms by which EVs contribute to disease development.

### Tumor-derived EVs

#### Tumor-derived EVs affect tumor cells and subpopulations.

Tumor-derived EVs are involved in the maintenance of cancer stem cells, metastasis, and resistance to chemotherapeutic drugs, and there is accumulating evidence of the direct and indirect roles of tumor-derived EVs in HMs. Tumor-derived EVs have been shown to directly modify the behavior of malignant cells in several types of HMs. For example, diffuse large B cell lymphoma tumor cell lines and patient samples were composed of flow cytometry–defined side population (SP) cells and non-SP cells. SP cells were characterized as weakly positive Hoechst 33342–stained cells that were postulated to be leukemia stem cells (LSCs) in HMs. The transfer of Wnt3a-containing EVs was involved in the cell state transition of non-SP into SP cells. Specifically, SP cells provided EV-Wnt3a to neighboring non-SP cells, resulting in activation of the canonical Wnt signaling pathway in recipient cells (37). EVs derived from a human chronic myeloid leukemia (CML) cell line (LAMA84) enhanced tumor growth both in vitro and in vivo by providing antiapoptotic molecules and TGF-β (38). Therefore, targeting of the EV autocrine effect is implicated as a potential therapy strategy. We also confirmed that blocking EV maturation and secretion by acute myeloid leukemia (AML) cells through *Vps33b* knockout/knockdown suppressed AML cell growth and prolonged disease progression in both a mouse model and patient samples (17). Similarly, lentivirus-mediated knockdown of *Rab27a* also decreased the EV levels and prolonged AML mouse survival (39). In addition, miR-34c-5p downregulated RAB27B, thus inhibiting the EV-mediated transfer and consequently increasing the senescence and eradication of LSCs through p53/p21/cyclin-dependent or p53-independent pathways (40). The EV-mediated autocrine effect was also observed in patient plasma. Comparison of the protein cargoes from indolent and progressive chronic lymphocytic leukemia (CLL) cells revealed that S100A9 protein levels in plasma EVs increased significantly with disease progression, thus contributing to disease progression via activation of the NF-KB pathway (41).

Collectively, these observations demonstrate the contribution of tumor-derived EVs to the organization of tumor cell populations and disease progression. The past decade has witnessed extraordinary advances in our understanding of how the interaction between cancer cells and their microenvironment contributes to disease progression and overall survival. As an important cell-cell communication mechanism, tumor-derived EVs were often found in these communication scenarios (42).

#### Effects of tumor-derived EVs on BM niche components.

**Figure 2:**
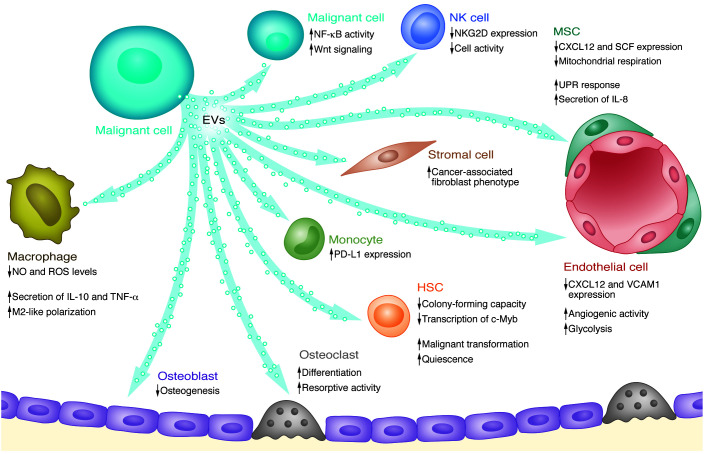
Roles and functional cargoes of tumor-derived EVs. Tumor-derived EVs, released by malignant cells, act as mediators of signals between malignant cells and hematopoietic cells (HSCs, macrophages, etc.) as well as non-hematopoietic cells (stromal cells, endothelial cells, osteoblasts, etc.). The crosstalk among various cell types via tumor-derived EVs results in remodeling the behaviors of recipient cells.

The BM niche appears to be remodeled in various HMs. The remodeled niche exhibits common features, such as increased hypoxia, angiogenesis, inflammation, and metabolic reprogramming (36). The concept of tumor-derived EVs as potent mediators of intracellular interactions is now widely accepted and has led to an increase in studies focused on deciphering these communication networks. Indeed, emerging evidence has confirmed that tumor-derived EVs actively contribute to formation of the tumor-permissive niche (Figure 2).

#### Endothelial cells.

Angiogenesis is a common feature of tumors. Tumor-derived EVs contribute to endothelial cell (EC) remodeling during angiogenesis in a variety of HMs. Multiple myeloma (MM) cells were found to regulate multiple pathways, resulting in increased BM EC line (STR10) viability, enhanced angiogenesis, and immunosuppression in a murine model, which further facilitated MM progression (43). MM-EV–contained Piwi-interacting RNA-823 (piRNA-823) was essential for the EC modulation required to support the growth of MM cells (44). Human AML cell–derived VEGF-containing EVs were responsible for glycolysis-mediated vascular remodeling and chemoresistance acquisition in AML (45). Furthermore, EVs derived from a CML cell line (LAMA84) induced a rapid reduction of CXCL12 and VCAM1 expression on ECs (46). Additionally, in acute promyelocytic leukemia (PML), EVs contained high levels of PML retinoic acid receptor-α transcripts. EV treatment resulted in the acquisition of procoagulant and tissue factor antigen in ECs (47). Since tumor blood vessels are key targets for therapeutic management, deciphering the mechanisms of EC remodeling in HMs is an important focus of research.

#### Mesenchymal stromal cells and descendant cells.

Experimental evidence has demonstrated that tumor-derived EVs can broadly modulate MSC proliferation, differentiation potential (mainly referring to osteogenesis and adipogenesis), hematopoietic supportive function, and metabolic profiling. In turn, these alterations modulated disease progression. AML-EV treatment increased Dickkopf-1 (DKK-1) expression and decreased osteogenesis of MSCs in an AML mouse model, providing direct evidence of the function of AML-EVs in vivo. More pertinently, leukemogenesis was largely accelerated after mice were pretreated with AML-EVs (39). AML-EVs also modulated the sensitivity of malignant cells to chemotherapy by altering MSC function. Mechanistically, AML-EVs elicited an unfolded protein response (UPR), which increased osteogenic priming of MSCs, potentially through the transfer of BMP2 (48). The UPR activated PERK/eIF2/ATF4 signaling during osteoblast differentiation followed by upregulation of the expression of genes that are essential for osteogenesis (49). Suppression of osteolineage cell function by tumor-derived EVs was also observed in MM and systemic mastocytosis (50–52).

In knockin syngeneic AML/acute lymphoblastic leukemia (ALL) mouse models, tumor-derived EVs increased the expression of adipose triglyceride lipase (ATGL) and hormone-sensitive lipase (HSL) enzymes in adipocytes, resulting in increased lipolysis, which supported leukemia cell expansion (53). Similarly, miR-92a-3p–containing EVs derived from a CML cell line (K562) attenuated adipogenesis of an adipose-derived MSC cell line (ADSCs) by inhibiting CCAAT/enhancer binding protein-α (C/EBPα). This inhibition of adipogenesis by tumor-derived EVs is postulated as a major mediator of cancer-associated cachexia (54).

EVs derived from primary AML patient cells and human leukemia/lymphoma cell lines (HEL, HL-60, MOLM-14, and U937) were internalized by a murine stromal cell line (OP9), resulting in increased proliferation and an altered growth factor secretion pattern in recipient cells. AML-EV–contained IGF-1R mRNA contributed to these changes (55). Furthermore, the same group demonstrated that AML-EVs downregulated critical retention factors (SCF, CXCL12) in stromal cells (56). Expression of JAG1 and SCF was also decreased after exposure to AML/myelodysplastic syndrome–derived EVs. This effect was partially abrogated by treatment with GW4869, which, as an inhibitor of the neutral sphingomyelinase SMPD2, blocks EV generation (57). Similar effects of tumor-derived EVs were found in other HMs (CML/CLL/MM/adult T cell leukemia/lymphoma [ATL]) (58–64). For example, CLL-EVs upregulated IL-8 expression in MSCs (60). Furthermore, miR-7977–containing EVs reduced the ability of human MSCs to support normal hematopoiesis via PCBP1 (57). Coincidentally, tumor-derived EV–contained miR-7977 modulated the Hippo/YAP signaling pathway in recipient MSCs, indicating its involvement in the increase in leukemia-supporting stroma growth (65).

Tumor-derived EVs can also regulate the metabolic state of BM stromal cells, which, in turn, become more supportive of malignant cells. Following internalization of ALL-EVs, a human stromal cell line (HS-5) showed a reduced oxygen consumption rate and increased extracellular acidification rate. These reprogrammed MSCs secreted an excess of lactate into the extracellular fluid, which is speculated to be the preferred energy source of tumor cells (66).

Taken together, these findings jointly illustrate that the modulations of MSCs caused by tumor-derived EVs not only constrain their capacity to support hematopoiesis, but also force them to become a shelter for leukemia cells.

#### Osteoclasts.

Reduced bone volume is a shared characteristic of multiple HMs. In addition to the reduction in osteolineage-forming cells, the recruitment and abnormal activation of osteoclasts also contribute to bone loss (67). Culturing with MM-derived EVs improved the migration and differentiation of primary human osteoclasts and increased expression of osteoclast markers (68). Treatment of mice with EVs derived from a murine MM cell line (5TGM1) promoted osteoclast formation and blocked osteoblast differentiation, which were attributed to the EV-contained DKK-1 protein. Intriguingly, GW4869-induced blockade of EV secretion not only increased cortical bone volume, but also sensitized myeloma cells to bortezomib (52). EV-contained EGFR ligand was subsequently shown to contribute to this phenomenon (69).

#### Fibroblasts.

The cancer-associated fibroblast is also an important niche component that is correlated with the survival of patients (70). Primary human myeloma cells modulated miR-27b-3p and miR-214-3p expression in fibroblasts through the release of EVs, which triggered proliferation and apoptosis resistance in myeloma fibroblasts via the FBXW7 and PTEN/AKT/GSK3 pathways, respectively (71). Shuttling of hTERT mRNA (the transcript of the telomerase enzyme) from Jurkat cells (human acute T lymphocyte leukemia cell line) via EVs transformed telomerase-negative fibroblasts into telomerase-positive cells, inducing increased proliferation, extension of lifespan, and the postponement of senescence (72). AML-EVs entered bystander fibroblast cells, resulting in increased proliferation and VEGF expression (55).

#### Macrophages.

M2-like macrophage induction and recruitment contribute to the formation of the immunosuppressive niche in tumors (73). Exposure to K562-derived EVs reduced NO and ROS levels in macrophages, and EV-treated macrophages were polarized to the M2-like phenotype, accompanied by elevated secretion of TNF-α and IL-10 (74). Furthermore, recent work confirmed that human primary MM cell–derived EVs also modulated the polarization toward M2-like macrophages. More importantly, abundant EV-contained miR-16 targeted the NF-κB canonical pathway, thus contributing to the M2-like macrophage polarization, and indicating that miR-16 overexpression represents a target for therapeutics with enhanced sensitivity (75).

In brief, these observations have shed light on the cellular components of the niche that are modulated by tumor-derived EVs; however, other noncellular niche components that are also modulated await further exploration. For instance, while tumor-derived EVs have been reported to be actively involved in matrix degradation in solid tumors (76), their participation in extracellular matrix remodeling in HMs remains poorly understood. Tackling this barrier would help to clarify the mechanisms underlying tumor infiltration and metastasis.

#### Tumor-derived EVs and normal HSPCs.

In many HMs, tumor cell infiltration is often accompanied by lethal cytopenia as a result of the impaired function of HSPCs. The profound suppression of HSPCs is caused not only indirectly by a less supportive niche (56, 57), but also directly through the action of tumor-derived EVs. The clonogenicity of HSPCs was attenuated by direct trafficking of AML-EVs containing microRNAs such as miR-150 and miR-155, which were sufficient to suppress murine HSPC clonogenicity, potentially by targeting the translation of the transcription factor MYB (77). We demonstrated that residual HSCs in leukemic mice were more quiescent than their counterparts in nonleukemic hosts (78). Later studies revealed that EVs impact the fate of HSCs via EV-dependent mechanisms. EV-contained miR-1246, which directly targeted the mTOR pathway and protein synthesis in HSCs, was shown to contribute to reversible quiescence and persistent DNA damage in murine HSCs (32). Similarly, AML-EVs carrying miR-4532 repressed normal hematopoiesis in human CD34^+^ HSPCs through activation of the LDOC1-dependent STAT3 signaling pathway (79). More importantly, hematopoietic progenitor cell differentiation was also compromised by EVs isolated from AML patient plasma through inhibition of dipeptidyl peptidase 4 (DPP4) in vitro (80). Therefore, exploring tumor-derived EV cargoes is likely to yield strategies that benefit hematopoietic regeneration and thus ameliorate cytopenia in HMs.

#### Tumor-derived EVs affect the immune niche and immunotherapy.

In addition to their capacity for niche modulation and HSPC repression, tumor-derived EVs have also been reported to contribute to immune suppression in various tumors (81). Here, we discuss whether and how tumor-derived EVs interfere with antitumor immunity in HMs. EVs released by B cell lymphoma cells carried CD20 that functions as a decoy target in rituximab treatment, thereby allowing cancer cells to escape treatment (82). On the other hand, EVs isolated from Burkitt’s lymphoma cell line (Jurkat and Raji cell) culture supernatants downregulated NKG2D receptor–mediated cytotoxicity and impaired NK cell function in vitro, thus indicating that EVs induced immune evasion in HMs (83). Moreover, EVs isolated from AML patient sera contained TGF-β1, membrane-associated major histocompatibility complex class I chain–related genes A/B (MICA/MICB), and myeloid blast markers, suggesting that they were probably secreted by leukemia blasts and potentially contributed to immune suppression. Confirmation that expression of the activating receptor NKG2D and NK cell activity decreased after treatment with AML serum–derived EVs further validated this hypothesis. More importantly, these impacts on NK cells were reversed by TGF-β1 neutralizing antibody treatment (84). The level of TGF-β1 in EVs might reflect a response to chemotherapy (85). In a phase I clinical trial, EVs isolated from AML patient sera blocked the antileukemia cytotoxicity and other functions of a human NK lymphoma cell line (NK-92), inducing the failure of adoptive cell transfer therapy (86). These observations indicated that removing EV-contained TGF-β would benefit immune restoration in patients. Interestingly, lentiviral shRNA-mediated silencing of TGF-β1 in both murine lymphocytic leukemia cell line (L1210) and secreted EVs reversed the immune repression effect in vitro and in vivo (87).

Advances have shown that immunotherapy is a promising approach for HMs, with several studies reporting that tumor-derived EVs can be successfully combined with adoptive T cell therapy. Tumor-derived EVs were internalized and presented by dendritic cells, inducing a potent CD8^+^ T cell–dependent antitumor effect on syngeneic and allogeneic murine tumors (88). The cytotoxicity of cytotoxic T lymphocytes was increased by exposure to leukemia-derived EVs that contained high levels of HSP70 and ICAM1, thereby enhancing leukemia antigen presentation (89, 90). However, in a later study, EVs were shown to induce immune escape by upregulating PD-L1 expression. Transcriptome and proteome analyses of human primary CLL-EVs revealed an abundance of noncoding Y RNA hY4, the transfer of which contributed to an increased release of CCL2, CCL4, and IL-6, as well as upregulating PD-L1 expression on monocytes (91). In particular, PD-L1–containing EVs from melanoma cells were sufficient to inhibit CD8^+^ T cells in vitro and in vivo, thus facilitating the progression of melanoma (92). Similarly, PD-L1–positive EVs from patient plasma induced T cell exhaustion after chimeric antigen receptor T cell therapy in CLL (93).

Immune therapy based on tumor-derived EVs is still in the proof-of-concept stage. Although tumor-derived EV molecules inherited from the parental cells could function as tumor-specific antigens, further profiling and investigation of their efficiency are required. The profound negative effects on immune cells and interference in immune therapy emphasize the importance of caution in the application of tumor-derived EV–based immune therapy.

### Niche cell–derived EVs

**Figure 3:**
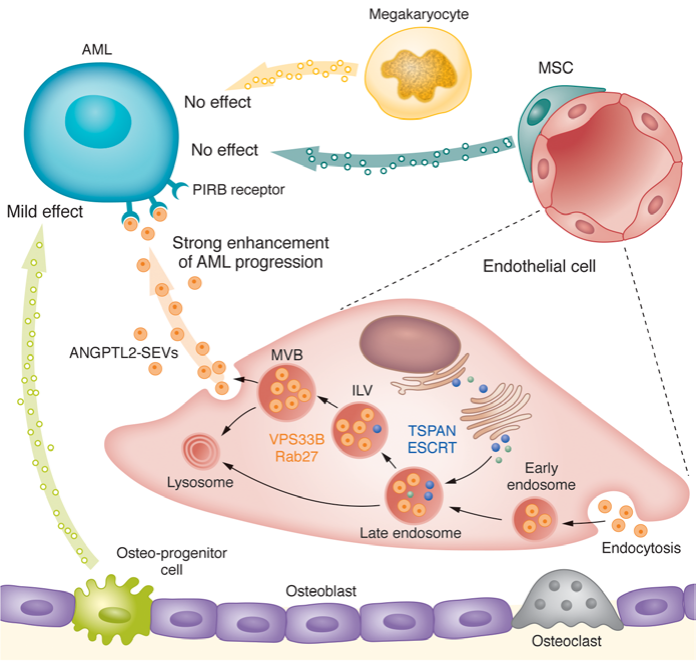
EVs derived from ECs accelerate the progression of AML. Various cellular components in the BM niche secrete EVs. Niche cell–specific conditional *Vps33b*-knockout mouse models confirmed that EC-derived EVs accelerated AML progression (17). EC-EVs contained a high level of ANGPTL2, which bound to the PIRB receptor on AML cells and further enhanced leukemia development via the p-SHP2/p-CREB pathway (100). MVB, multivesicular body; ILV, intraluminal vesicle; TSPAN, tetraspanin; ESCRT, endosomal sorting complex required for transport; SEV, small extracellular vesicle.

Given that cell-cell communication is a “two-way street,” researchers have focused on deciphering the roles of EVs derived from certain niche cell types. In a CML mouse model, miR-126 was transferred from ECs to CML-LSCs via EVs. Furthermore, conditionally knocking out miR-126 from ECs delayed leukemia progression and improved survival (94). However, the extent to which EV–miR-126 contributes to the overall transfer of miR-126 is unknown. In addition, in vitro cultures indicated that MSC-EVs protected AML cells against the cytotoxic effects of tyrosine kinase inhibitors (95, 96). EVs derived from the MSCs of primary MM patients (MM BM MSCs) and healthy volunteers (BM MSCs) caused opposing effects on tumor growth when transferred to MM cells, as MM BM MSC-EVs were found to promote MM tumor growth while BM MSC-EVs inhibited growth. It can be speculated that these opposing effects can potentially be explained by differences in the contents of microRNAs (miRNAs) and oncogenic proteins in the EVs (97). Another study showed that BM MSC-EVs increased proliferation and drug resistance in human MM cells (98). The uncertainty and contradictory conclusions among studies of EVs are an inevitable result of not only the heterogeneity of niche cells, but also differences in experimental factors such as models or culture conditions, EV isolation methods, doses, and intervals of administration. Genetically manipulated animal models are useful for clarifying these contradictions and systemically analyzing the function of specific cell type–derived EVs in vivo (99). We recently conducted a systematic exploration of the effects of specific BM niche cell–derived EVs using a conditional *Vps33b*-knockout mouse model and showed that EC-EVs accelerated AML progression. Mechanistically, we found that EC-EVs contained a high level of ANGPTL2, which bound to the PIRB receptor on AML cells and enhanced leukemia development. Furthermore, blocking the secretion of ANGPTL2-containing EVs from ECs delayed the progression of AML (Figure 3) (100). Thus, our research indicates the value of conditional knockout mouse models for exploring cell type–specific EV function in other systems and conditions, leading to a deeper understanding of the physiological and pathological roles of EVs.

## Clinical applications

### EVs as biomarkers

EVs can be detected in almost all kinds of biological fluids (101). Multi-omics readouts of these EV cargoes would offer insights into the functional state of the tissues and organs, providing signals that can be used to monitor disease burden and predict prognosis.

Elevated levels of EVs and distinct molecular profiles have been identified in various HMs (84, 102, 103). More importantly, changes in EV levels are correlated with fluctuations in tumor burden (85, 104). Increased EV levels were detected in an AML patient–derived xenograft mouse model, and notably, EVs collected from the recipient mice faithfully mimicked the molecular features of those from patients (105). EV provides a protective “shelter” against degradation of RNA by RNase A, thus becoming a source of enriched miRNAs (106). A study about the feasibility of measuring EV-miRNA to monitor minimal residual disease (MRD) in AML patients showed that a set of miRNAs were enriched in circulating EVs and could be used to distinguish leukemia xenografts from healthy human CD34^+^ cells. These EV-miRNAs were detected in patients with low BM tumor burden before circulating blasts were generated (107). As such, this study provided proof-of-concept evidence of the utility of EV-miRNAs for monitoring MRD.

Distinct EV-miRNA signatures have been observed in clinical studies. MiR-150, miR-155, and miR-29 were upregulated and miR-223 was downregulated in CLL plasma–derived EVs (103). MiR-155 was also elevated in EVs derived from the serum of patients with AML and Waldenström’s macroglobulinemia (108). Two studies have indicated the potential value of EV-miRNAs as biomarkers of human BM failure diseases. By screening of the miRNA profiles of plasma EVs, 25 differentially expressed miRNAs were identified in aplastic anemia (AA) and/or myelodysplastic syndrome, among which miR-126-5p was negatively correlated with response to immunosuppressive therapy in AA patients (109).

Both the plasma-circulating molecules and those packaged in EVs may serve as potential biomarkers; however, RNA-Seq data revealed distinctive RNA profiles of EVs and showed homogeneity in the RNA compared with the total plasma contents. These findings indicate that the profiles of circulating miRNAs and EV-miRNAs represent distinct snapshots of disease (110). EVs could also serve as new tools for disease diagnosis. CD34^+^CD71^lo^ EVs were reported as alternative indicators in the diagnosis of inherited Diamond-Blackfan anemia (DBA), as an absence of these EVs was associated with a low level of erythroid burst–forming units in DBA patients (111). Nevertheless, the clinical potential of EV profiles should not be overestimated until standard protocols have been established for evaluation, isolation, and assessment. The sensitivity and specificity of applying EV cargoes as biomarkers also need further exploration (112).

### Targeting tumor-derived EVs in therapeutic regimens

Tumor-derived EVs enhance tumor cell survival by promoting the formation of a permissive niche or repressing antitumor immune attack, indicating their great potential as therapeutic targets. Blocking EV secretion from tumor cells improved the survival of AML mice in several models (17, 40, 100). However, this approach is limited by the challenge of specifically blocking the EVs derived from a certain cell type. As EVs can be derived from many cell types, targeting of EV biogenesis should be approached with caution (113). In practice, clinical benefit does not necessarily depend on absolute EV blockade, as targeting certain tumor-derived EV–enriched molecules would also improve symptoms or overall survival.

### EVs as a cell-free treatment

EVs represent a cell-free replacement for cell therapy, especially when combined with genetic engineering tools; however, the efficacy of the cell-derived EVs is a prerequisite of this application. EVs derived from induced pluripotent stem cells or young MSCs reduced cellular ROS levels and alleviated aging phenotypes of senescent MSCs (114, 115). EV-based therapy has also shown promising efficacy in clinical trials. In particular, based on an array of integrated analyses, including cell culture systems, mouse models, and clinical trials, MSC-EV administration in graft-versus-host disease (GVHD) was shown to offer a paradigm for EV-based cell-free therapy. MSC-EVs isolated from unrelated healthy donor BM contained a quantity of antiinflammatory molecules, but not proinflammatory cytokines and apoptosis-inducing molecules (116). Moreover, the symptoms of GVHD patients were significantly improved by MSC-EV administration, implying that BM MSC-EVs have important therapeutic potential in GVHD (117). In accordance with clinical cases, GVHD model mice treated with human CB MSC-EVs exhibited a reduced immune response and improved survival (118). Amelioration of GVHD after infusion with human BM MSC-EVs was associated with circulating T cell preservation. Microarray analysis of MSC-EVs identified that miR-125a-3p was a potential candidate in this process (119). In a phase I study, clinical improvement was observed in 70% of high-risk or steroid-refractory acute GVHD patients after infusion of Wharton’s jelly–derived MSCs (WJMSCs) (120). WJMSCs significantly increased circulating PD-L1–positive EVs, and PD-L1 was essential to T cell function suppression (121). These reports of the tolerance and improvement of GVHD symptoms induced by MSC-EVs inspired a phase II clinical trial of the treatment of chronic GVHD patients with umbilical MSC-EVs (ClinicalTrials.gov NCT04213248).

### Engineering EVs for drug delivery

Owing to their biocompatibility, stability, and limited immunogenicity, EVs provide multiple advantages as a delivery system over traditional synthetic delivery vehicles (122). For example, due to their virus-like diameters, and their capacity to be recognized and internalized by specific recipient cells, EVs can target lesions in anatomically isolated compartments, such as the central nervous system (123). It has been confirmed that modified EVs can cross the blood-brain barrier to deliver siRNAs (124). Modification strategies to achieve effective delivery of therapeutic vehicles have been widely explored. These strategies can be broadly classified as surface chemistry approaches and genetic engineering approaches (125). RBC-derived EVs (RBC-EVs) can be modified by the addition of a linker peptide through a combination of enzymatic ligation and streptavidin-biotin conjugation. These modified EVs facilitated accumulation of RBC-EVs in metastatic cancer cells, leading to potent tumor-specific CD8^+^ T cell immune response, which contributed to a prominent suppression of breast cancer metastasis in the lung (122). Impressive research has yielded effective methods for genetic engineering of EVs. For example, engineered EVs have been shown to target oncogenic substances and suppress cancers (126). Subsequently, the same group established good manufacturing practice–compliant procedures for producing a clinical-grade product (127). This ground-breaking study illustrated the process and feasibility of generating clinical-grade EVs for various therapies of human diseases, thereby paving the way for further clinical application. Although still in its infancy, EV-mediated drug delivery has clearly shown great potential as cell-free therapies in a wide range of diseases.

## Conclusions and research prospects

In this Review, we have discussed evidence for the roles of EVs in normal hematopoiesis and HMs, focusing on the function of specific cell-derived EVs and their cargo molecules. In normal hematopoiesis, EVs regulate the “SMART” properties of HSCs. In HMs, tumor-derived EVs and normal cells (hematopoietic or non-hematopoietic) engage in mutual crosstalk, resulting in disease progression.

However, although increasing lines of evidence indicate that EVs play important roles in normal hematopoiesis and hematopoietic disorders, a limitation of many EV studies so far is their reliance on in vitro experiments. More efficient in vivo models are required to fully elucidate the mechanisms related to the formation and functions of EVs. The use of genetic tools to specifically abrogate EV release or label EV transfer to a given cell type without substantially interfering with other biological process could be the most robust approach to more informative explorations in less perturbed and unbiased assays.

To date, most studies have focused on EVs derived from different cells and their effects on hematopoietic homeostasis. However, several key issues related to the biological behaviors of EVs remain to be addressed. For instance, the mechanisms underlying EV biogenesis await investigation. Although many Rab family members have been reported to be involved in sorting proteins into vesicles, the processes that lead to EV maturation, cargo loading, and transfer to individual EVs from different cell types, and the generation of EVs, are still unclear. In addition, the mechanisms by which EV components affect HSPCs or LSCs may vary because of their specific localizations in EVs, such as on the membrane or inside EVs. The sources of EVs from niche components or other tissues may also interact to influence HSPC or LSC activities. Therefore, delineation of the effects and mechanisms of EVs in normal hematopoiesis and hematopoietic disorders, especially in the hematopoietic ecosystem in vivo, remains a future challenge and warrants extensive studies.

## Figures and Tables

**Table 1:**
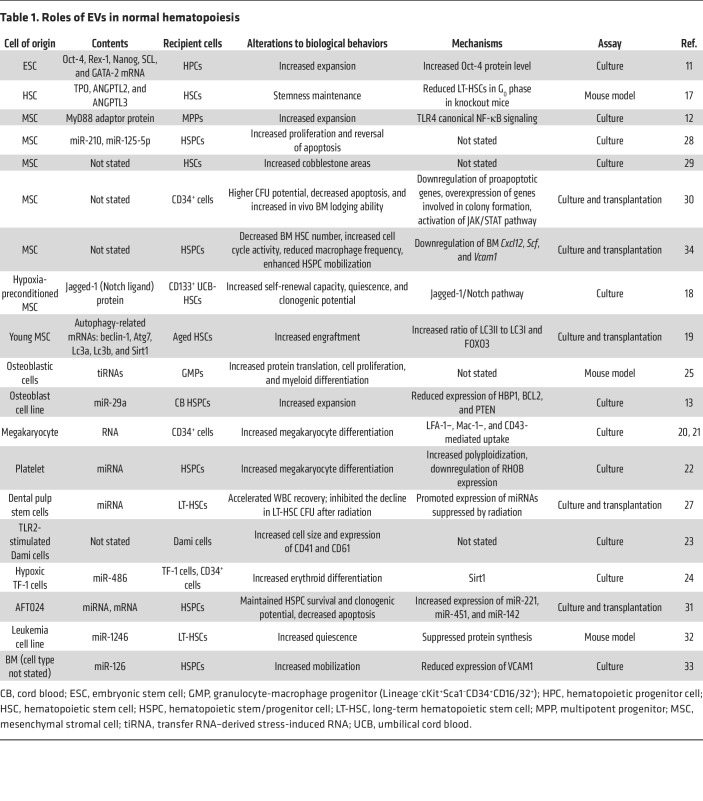
Roles of EVs in normal hematopoiesis

## Version 1 09/15/2022

Electronic publication
